# Neural changes in early visual processing after 6 months of mindfulness training in older adults

**DOI:** 10.1038/s41598-020-78343-w

**Published:** 2020-12-03

**Authors:** Ben Isbel, Jan Weber, Jim Lagopoulos, Kayla Stefanidis, Hannah Anderson, Mathew J. Summers

**Affiliations:** 1 Thompson Institute, University of the Sunshine Coast, Locked Bag 4 (ML59), Maroochydore DC, QLD 4558 Australia; 2 Graduate Training Center of Neuroscience, International Max Planck Research School, University of Tuebingen, Österbergstrasse 3, 72074 Tuebingen, Germany; 3 School of Health and Behavioural Sciences, University of the Sunshine Coast, Locked Bag 4 (ML59), Maroochydore DC, QLD 4558 Australia

**Keywords:** Cognitive neuroscience, Cognitive ageing

## Abstract

Mindfulness has been shown to improve attentional performance, which is known to decline in aging. Long-latency electroencephalographic (EEG) event-related potential (ERP) changes have been reported immediately after mindfulness training, however the enduring stability of these effects is unknown. Furthermore, the ability of mindfulness to impact earlier stages of information processing is unclear. We examined neural activation using high density EEG in older adults engaged in mindfulness training to examine the long-term stability of training effects. After 6 months of training, mindfulness practitioners displayed enhanced neural activation during sensory encoding and perceptual processing of a visual cue. Enhanced perceptual processing of a visual cue was associated with increased neural activation during post-perceptual processing of a subsequent target. Similar changes were not observed in a control group engaged in computer-based attention training over the same period. Neural changes following mindfulness training were accompanied by behavioural improvements in attentional performance. Our results are suggestive of increased efficiency of the neural pathways subserving bottom-up visual processing together with an enhanced ability to mobilise top-down attentional processes during perceptual and post-perceptual processing following mindfulness training. These results indicate that mindfulness may enhance neural processes known to deteriorate in normal aging and age-related neurodegenerative diseases.

## Introduction

An emerging body of research suggests that mindfulness training may enhance attentional performance through the repeated activation of neural circuitry associated with information processing and attentional control^1^. Mindfulness training involves the development of sustained attention via the repeated activation of metacognitive monitoring and control processes to resist distraction from an object of focus, which for beginners is commonly a localised set of sensations accompanying the breath^2^. This style of attention training is coupled with an equanimous attitudinal orientation toward the contents of experience. Equanimity supports the development of attentional stability by reducing both cognitive and affective responses which might otherwise disrupt attention. When attention wanders, practitioners are required to inhibit and disengage from distracting processes and redirect attention back toward the breath, thereby repeatedly activating attentional control processes. Accordingly, mindfulness interventions have been shown to improve sustained attention^3,4^, selective attention^5,6^, and executive inhibitory control^7,8^ and increase activation in neural circuits associated with attentional deployment and control located in the anterior cingulate cortex^1^ and right dorsolateral prefrontal cortex^9^.

Electroencephalographic event-related potentials (ERP) generated by spatially aligned cortical pyramidal cells provide sensitive temporal measures of information processing in the human brain and are ideally suited to the investigation of attention^10^. The time course of visual processing in the human cortex can be traced by ERP responses elicited during sensory encoding (50-80 ms), early stimulus discrimination (100–200 ms), and later cognitive processing (200–400 ms). The extent to which mindfulness is capable of exerting effects on these different stages of information processing is unknown. A handful of studies have provided evidence of changes to long-latency N2 and P3 ERP components following mindfulness training indicative of faster deployment and enhanced allocation of attentional resources during controlled cognitive processing^11–14^. These effects suggest that the attention training component of mindfulness practice is capable of modifying top-down attentional processes. While long-latency ERP effects have been reported following 8 weeks of mindfulness training, little is known regarding the stability of these effects beyond a short-term intervention. Two cross-sectional studies comparing long-term practitioners with matched controls report neural changes in short-latency P1 and N1 ERP components in addition to N2-P3 changes, suggesting that with prolonged practice mindfulness may exert up-stream effects on earlier stages of information processing^5,15^. This ability to deploy greater attentional capacity at earlier stages of information processing is predicted by contemporary cognitive models of mindfulness which assert that increased attentional capacity may be allocated at earlier and earlier stages of information processing as proficiency in this style of attention training grows^2^. The observation of both short and long-latency ERP effects in long-term practitioners and only long-latency effects in those completing an 8-week intervention supports the thesis that mindfulness may gradually exert earlier effects on the temporal sequence of information processing as proficiency increases. However, to date no longitudinal study has been conducted to examine the training trajectory of these ERP changes.

Perceptual and attentional processes are generally thought to be capacity limited and are known to slow and decline with age^16^. Age-related slowing of visual processing has been shown to result from deterioration in the white matter microstructure of the optic radiation connecting the thalamus to the visual cortex, specifically in the magnocellular pathway^17,18^. Deterioration in the neural pathways associated with bottom-up visual processing is further impacted by diminished top-down attentional facilitation of sensory input with increasing age, resulting in longer latency and reduced amplitude of the visual P1 and N1 ERP components^19^. Furthermore, greater susceptibility to distraction as a result of deteriorating inhibitory control is a characteristic feature of age-related cognitive decline and neurodegenerative disease, resulting in reduced sustained attention with increasing age^20,21^. These age-related declines in top-down attentional performance are characterised by increased latency and reduced amplitude of long-latency N2 and P3 ERP components which index subsequent cognitive processing of previously encoded information^10^. Mindfulness preferentially trains executive attentional control processes such as inhibitory control to develop sustained attention to present-moment experience^2^, leading to the enhanced attentional and neurophysiological changes discussed above. While this ability to improve attentional processes susceptible to aging suggests a potential role for mindfulness in treating age-related and neurodegenerative cognitive declines, few studies have investigated the efficacy of mindfulness in older adults.

It should be noted that cognitive training interventions have also been shown to be capable of modifying long-latency ERP components and improving attentional performance in older adults. In a comparison of training interventions to improve cognitive function in healthy older adults, cognitive training was found to improve attentional performance and increase N2 and P3 amplitudes^22^. No such changes were observed in either a physical training or relaxation condition, suggesting that cognitive training was capable of producing both behavioural and neural changes indicative of improved attentional performance in healthy older adults. Similar cognitive training-related improvements to working memory, executive function, and processing speed have previously been reported in older adults^23,24^, and these improvements have been accompanied by increases in N2 and P3 amplitudes^25–27^, indicating an enhanced ability to deploy attentional resources after training. While these ERP amplitude changes have been widely reported in older adults following cognitive training programs, there is no evidence of their ability to alter ERP component latency^28^.

Attention training in mindfulness is similar to attention training as operationalised in cognitive training programs. It remains unknown if the neurophysiological changes arising from these two types of intervention result from this shared attention training component. It should be noted that in mindfulness this shared attentional component is conjoined with the cultivation of an equanimous attitudinal approach to the contents of experience, and in contrast to cognitive training programs, short-term mindfulness training interventions have been shown to alter both the amplitude and latency of late ERP components in older adults^11,13,29^. Given that both cognitive models of mindfulness and cross-sectional evidence from long-term mindfulness practitioners suggest that these short-term neurophysiological effects may be capable of appearing earlier in the temporal sequence of information processing with increasing proficiency in the practice, longitudinal studies assessing temporal change beyond immediate intervention effects are required.

We conducted an active controlled longitudinal RCT in healthy older adults to assess both the immediate neurophysiological effects of an 8-week mindfulness intervention (T1–T2) together with a follow-up assessment 6 months after the commencement of training (T3) to assess whether training effects appeared earlier in the temporal sequence of information processing with continued practice. The mindfulness training (MT) condition utilised a standardised mindfulness technique developed for use in longitudinal RCTs^2^. Previous studies have commonly utilised therapeutic mindfulness-based interventions (MBIs) such as either mindfulness-based cognitive therapy (MBCT)^30^ or mindfulness-based stress reduction (MBSR)^31^ as intervention conditions. These multifaceted MBIs were developed for use in therapeutic settings. As a result, they combine mindfulness training with adjunct practices including yoga, relaxation, and therapeutic techniques, which may undermine their suitability as a training condition when seeking to assess the effects of mindfulness alone. Importantly, the MT condition utilised in the current study operationalises mindfulness without the inclusion of ancillary components, thereby rendering it suitable to make clear causal inferences regarding observed outcomes.

Event-related potentials were elicited using an AX-CPT variant of a continuous performance task known to assess sustained attention^32^. Participants are required to attend to the rapid serial presentation of letters on a computer screen and respond by keypress when they observe a target letter ‘X’, but only when that ‘X’ is preceded by a cue letter ‘A’. Errors on this task index gross failures of sustained attention whereas reaction time co-efficient of variation (RTCV) indexes stability of attentional deployment, where greater fluctuations in sustained attention are observed as increased variability of responding^33^. The AX-CPT has been widely used in studies of cognition and is capable of detecting changes in the neural circuits underlying attentional deployment and control. Performance on the AX-CPT is directly related to the extent of any impairment within these neural networks regardless of etiology^34^.

A computer cognitive training (CT) control condition designed to activate similar attentional processes to those activated during mindfulness was used to determine if the neurophysiological effects of mindfulness could be explained by its attention training component. As mindfulness recruits neural networks associated with sustained attention, selective attention, executive control, and working memory, a game-based format presenting modified versions of standard psychological tasks designed to activate the same attentional processes was utilised in the CT condition. Participants in the CT program were instructed to perform the same task continuously each session to replicate the training of sustained attention that occurs in mindfulness practice, and tasks were changed weekly to simulate the changing attentional demands that occur throughout a mindfulness training intervention. To validate the effectiveness of the interventions, a breath counting task^35^ was used as a behavioural measure of trait mindfulness to confirm that the MT condition was successful at improving mindfulness, while the CT condition was able to train attention without improving mindfulness.

In line with evidence demonstrating attentional improvements and long-latency ERP changes resulting from both cognitive training and mindfulness interventions, we hypothesised that both the MT and CT conditions would enhance attentional performance and effect long-latency ERP changes immediately following the 8-week training period. Furthermore, if the attentional component of mindfulness is responsible for the reported ERP changes resulting from this practice then both the MT and CT conditions should result in similar ERP changes, since the CT condition was designed to replicate the type of attentional training found in the MT condition. Lastly, in line with both the predictions of the cognitive models of mindfulness and the cross-sectional evidence suggesting that with increasing proficiency mindfulness may impact earlier stages of information processes, we hypothesised that ERP changes in the MT group would shift earlier in the temporal stream of information processing after 6 months of training.

Since we were interested in examining ERP effects predicted to be distributed over a broad time range and electrode distribution, a non-parametric cluster-based permutation analytic approach was chosen^36^. This analytic technique is driven by a neurophysiologically informed approach to data handling that incorporates both temporal and spatial considerations, permitting the examination of spatiotemporal ERP dynamics while at the same time elegantly controlling the family-wise error rate^37^. In contrast to conventional ERP analyses where the main observations (e.g. mean or peak amplitude of a certain component) are extracted within a priori defined time windows and electrodes, cluster-based permutation statistics quantify the statistical effect between the conditions of interest at every time-sensor pair at each data point in the time series. Since the aim of the present study was to investigate the currently unknown temporal effects of mindfulness training on information processing, we decided to use non-parametric cluster-based permutation statistics as they provide an elegant way to unravel new insights into the spatiotemporal dynamics of both early and late stages of information processing.

## Results

No significant pre-intervention differences between groups were observed in age, gender balance, estimated full-scale intelligence quotient, handedness, breath-counting task accuracy, or AX-CPT performance (see Table 1).

**Table 1:** Pre-intervention participant characteristics by group.

Baseline characteristics		MT group (*n* = 50)	CT group (*n* = 31)	Statistics
**Demographics**				
Age (years)	*M* (SD)	71.9 (4.8)	69.6 (5.9)	*t*_*(79)* =_ 1.94, *p* = .056
Gender	(% female)	58.8%	70%	χ_(1*, N* = 81)_ = 1.01, *p* = .314
Predicted FSIQ	*M* (SD)	112.7 (7.0)	112.5 (6.6)	*t*_*(79)* =_ 0.11, *p* = .912
Handedness	(% RH)	96.1%	93.3%	χ_(1*, N* = 81)_ = 0.30, *p* = .582
**Behavioural data**				
Breath-counting accuracy (%)	*M* (SD)	44.8 (32.7)	53.4 (34.8)	*t*_*(79)* =_ 1.12, *p* = .267
AX-CPT RT (ms)	*M* (SD)	433.3 (48.6)	436.3 (56.9)	*t*_*(79)* =_ 0.25, *p* = .807
AX-CPT RT CV	*M* (SD)	0.14 (0.04)	0.14 (0.05)	*t*_*(79)* =_ 0.43, *p* = .670
AX-CPT total errors (%)	*M* (SD)	0.45 (0.51)	0.39 (0.52)	*t*_*(79)* =_ 0.50, *p* = .621

*FSIQ* full scale intelligence quotient, *RH* right-handed, *RT* reaction time, *RT CV* reaction coefficient of variation.

### Intervention training time

**Figure 1:**
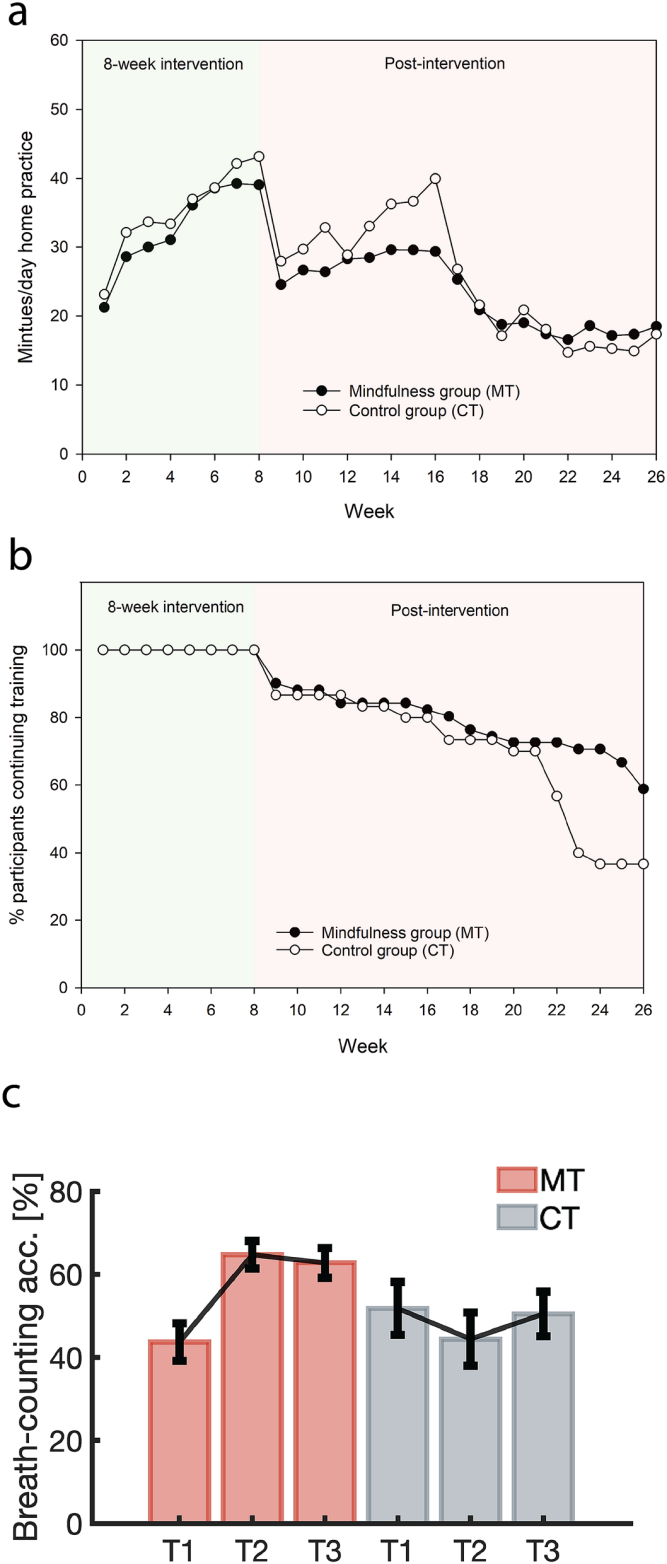
Intervention engagement and mindfulness scores over the 6-month study period. (**a**) Weekly average of minutes/day home training times over the course of the 8-week intervention and post-intervention period. (**b**) Training exercise participation rates over the course of the study period. (**c**) Breath counting accuracy scores over the three timepoints. Error bars show SEM.

Inspection of participant training records over the 8-week intervention showed that the CT group [*M* = 35.4 min/day (*SD* = 5.5)] spent approximately two minutes longer than the MT group [MT: *M* = 33.0 min/day (*SD* = 4.2)] training each day during the program (*t*_(79)_ = 2.21, *p* = 0.030). At 6-month follow-up, 36.7% of CT and 58.8% of MT participants reported some level of continuing engagement with the training exercises. See Fig. 1a for daily training times and Fig. 1b for continuing participant engagement over the 6-month study period. When averaged over the 6-month period, the CT group [*M* = 30.1 min/day (*SD* = 6.0)] spent approximately three minutes per day longer than the MT group [*M* = 27.2 min/day (*SD* = 5.4)] engaged in the training exercises (*t*_(69)_ = 2.29, *p* = 0.025).

### Change in mindfulness

A breath counting task was used to assess the effectiveness of the MT intervention to enhance mindfulness skills in this mindfulness-naïve sample. Breath counting has been validated as a behavioural measure of mindfulness^35,38^ and has recently been shown to demonstrate greater discriminant validity than self-report measures in assessing changes in mindfulness^39^. Mixed model ANOVA of breath counting scores revealed a significant group × time interaction (*F*_(2,154)_ = 6.95, *p* = 0.001, *d* = 0.60; see Fig. 1c). Follow-up pairwise comparisons showed that the MT group demonstrated significant improvements from T1 to T2 (t_(49)_ = 4.2, *p* = 0.0001), and these gains were retained at 6-month follow-up (T1–T3 pairwise comparison: t_(49)_ = 3.2, *p* = 0.002). No significant change in breath counting scores was observed in the CT group across the three time-points. These findings demonstrate that the MT intervention was successful at developing mindfulness skills, while the CT condition did not improve mindfulness.

### Change in attentional performance

**Figure 2:**
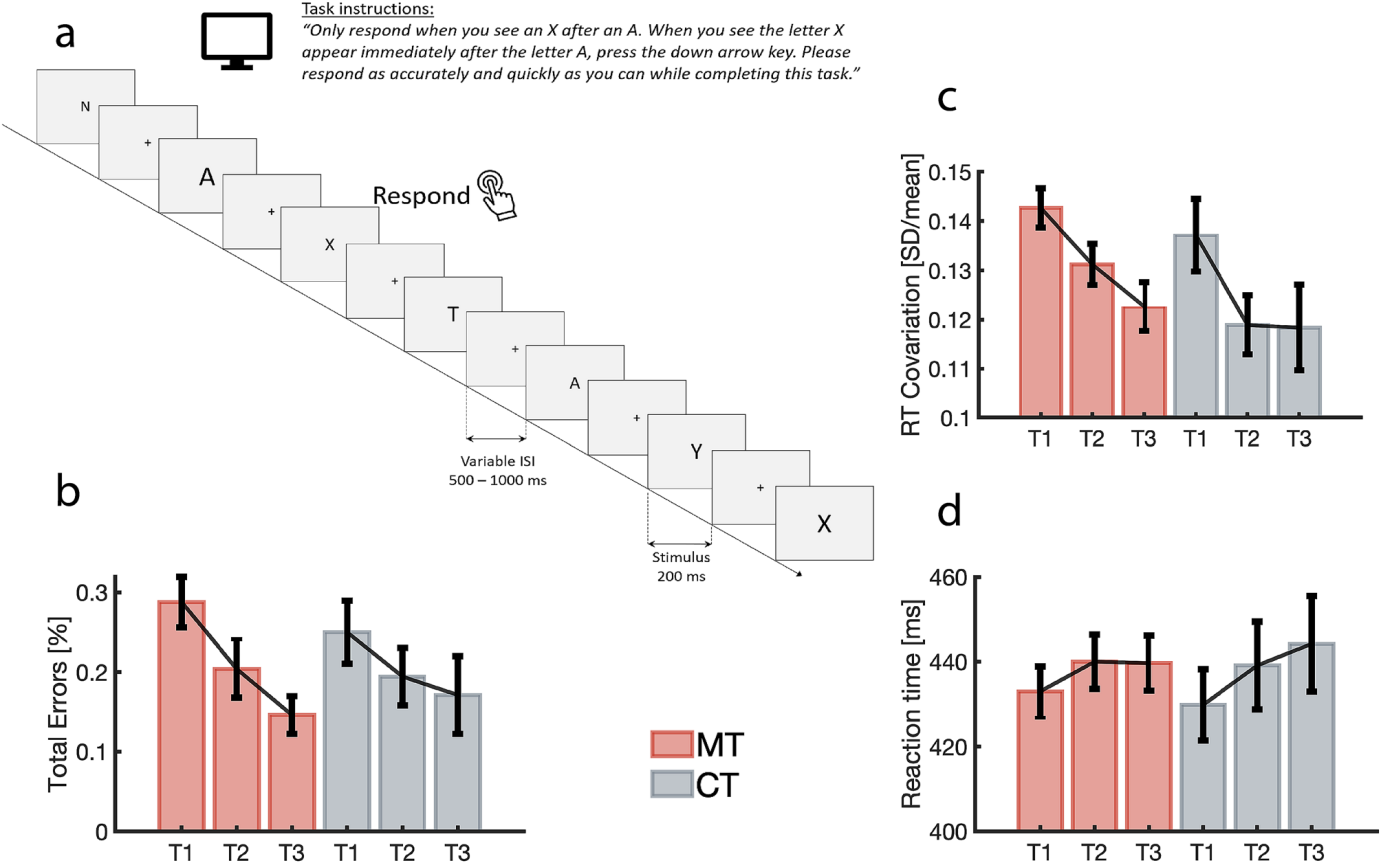
AX-CPT experimental design and behavioural results. (**a**) AX-CPT task design. Following a variable inter-stimulus interval (500–1000 ms) during which participants focussed upon a central cross, angular shaped letters (A, E, F, H, L, N, T, V, X, Y, Z) in a randomly selected font size (100–180 point) were presented at the central location for 200 ms. Participants were required to respond as quickly and accurately as possible via keypress only when they observed the letter X immediately following the letter A. (**b**) Group average total AX-CPT errors over time. Errors bars show SEM. (**c**) Plots of reaction time coefficient of variation (RTCV) by group over time. Error bars show SEM. (**d**) Plots of reaction time (RT) by group over time. Error bars show SEM.

Performance measures on the AX-CPT (see Fig. 2a) were used to assess attentional outcomes of the two programs. Since both the MT and CT conditions were attention training interventions emphasising the development of sustained attention, we expected both groups to demonstrate improved attentional performance on the AX-CPT. In line with this prediction, mixed model ANOVA revealed a significant effect of time for AX-CPT errors (*F*_(2,132)_ = 11.64, *p* < 0.0001, *d* = 0.85; Fig. 2b) and RTCV (*F*_(2,144)_ = 11.79, *p* < 0.0001, *d* = 0.81; Fig. 2c), while no significant change in RT was observed (*F*_(2,144)_ = 2.87, *p* = 0.06, *d* = 0.40; Fig. 2d). No significant group × time interaction (*p* > 0.05) was observed on any AX-CPT measure, indicating that error rates decreased across both groups over time, while the stability of attentional deployment improved across both groups. Exploratory pairwise comparisons for AX-CPT errors showed that while both groups made fewer errors over time, the MT group demonstrated significant reductions in errors from T1 to T2 (t_(43)_ = 3.57, *p* = 0.0004, Hedge’s g = 0.65), and from T1 to T3 (t_(43)_ = 4.26, *p* = 0.00006, Hedge’s g = 0.82), while the CT group demonstrated no significant change in errors from either T1 to T2 (t_(23)_ = 1.08, *p* = 0.14, Hedge’s g = 0.26) or from T1 to T3 (t_(23)_ = 1.40, *p* = 0.09, Hedge’s g = 0.27).

Taken together the behavioural results demonstrate that both the MT and the CT conditions successfully improved attentional performance as indexed by AX-CPT error rates and RTCV. Improvements in breath counting accuracy were observed only in the MT group, demonstrating that while the MT condition improved both attention and mindfulness, the CT condition was successful at improving attention without improving mindfulness. Significantly, immediate training effects were retained at 6-month follow-up.

### Change in event-related neural activity

High-density EEG obtained during the AX-CPT was analysed to assess neurophysiological changes resulting from the training programs. Separate ERP analyses were conducted for the cue-stimulus A and the target-stimulus X of the sequential AX pair. To assess neurophysiological changes, we applied the following analysis strategy: In a first step, we calculated main factor effects (group = MT vs. CT and time = T1 vs. T2 vs. T3) as well as interaction effects. Secondly, we computed follow-up post-hoc statistics arising from step one. It is important to note here that aside from the strengths and advantages of using a cluster-based permutation analytic approach (e.g. elegantly correcting for multiple comparisons), difficulties arise when applying this analytic approach to test interaction effects when one experimental factor has more than two levels. As this is the case in our experimental design (three levels for factor time [T1/T2/T3]), we attempted to assess pairwise interaction effects by means of contrasting the difference between two timepoints, e.g. ([MT T3 − MT T1] − [CT T3 − CT T1]) for testing a group × time interaction effect between T1 and T3^40^.

**Figure 3:**
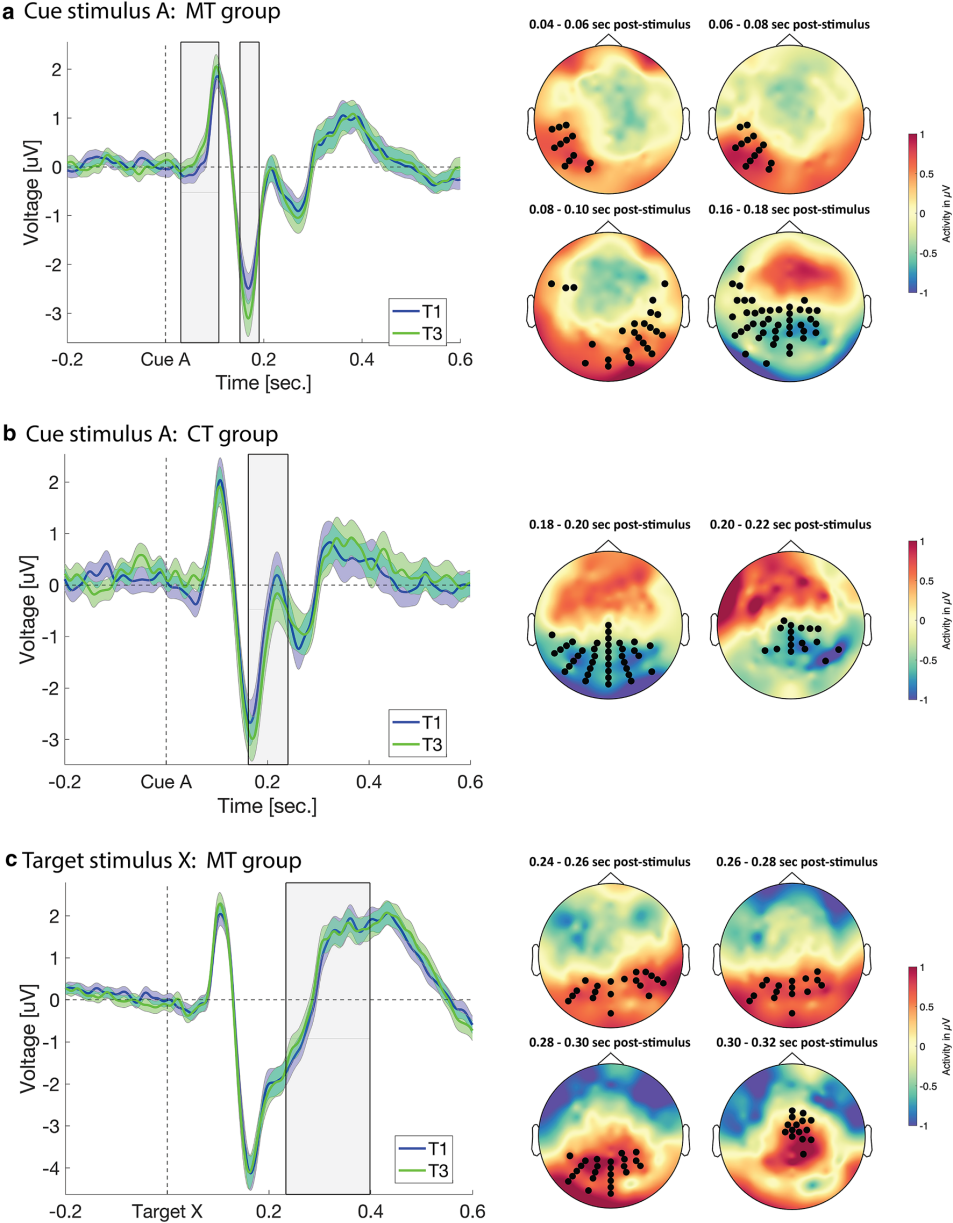
Event-related activity cluster analysis. (**a**) Cue stimulus ERP cluster analysis (with shaded SEM) and topography for the MT group. Shaded boxes show significant difference cluster ranges over time. Topographic maps show significant cluster scalp distribution over time. (**b**) Cue stimulus ERP cluster analysis and topography for the CT group (with shaded SEM). Shaded box shows significant difference cluster range over time. Topographic maps show significant cluster scalp distribution over time. (**c**) Target stimulus ERP cluster analysis (with shaded SEM) and topography for the MT group. Shaded box shows significant difference cluster range over time. Topographic maps show significant cluster scalp distribution over time.

**Figure 4:**
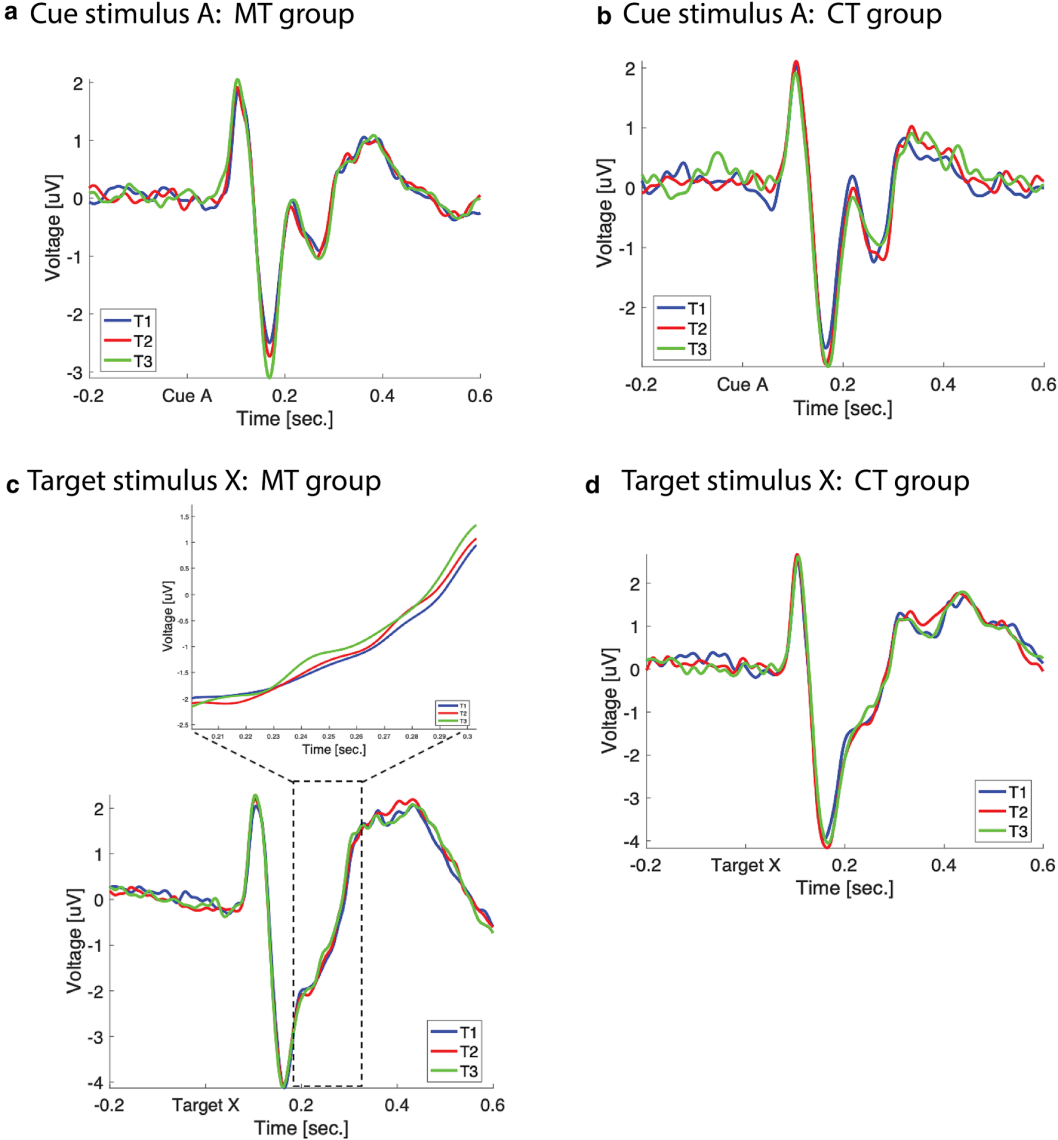
ERP plots for all three timepoints for each stimulus type and group. (**a**) ERP waveforms for the MT group in response to the cue stimulus for all timepoints. (**b**) ERP waveforms for the CT group in response to the cue stimulus for all timepoints. (**c**) ERP waveforms for the MT group in response to the target stimulus for all timepoints. Expanded view shows ERP waveforms during the significant cluster period. (**d**) ERP waveforms for the CT group in response to the target stimulus for all timepoints.

A non-parametric cluster-based permutation test on the trial-averaged ERPs in response to the cue-onset (stimulus A) revealed an overall main effect of time (cluster 1: sum(*F*) = 1.76 × 10^4^, *P*_cluster_ = 0.004; cluster 2: sum(*F*) = 1.22 × 10^4^, *P*_cluster_ = 0.021). However, no main effect of group was observed (maximal cluster: sum(*t*) = 1.26 × 10^3^, *P*_cluster_ = 0.58) and no significant pairwise difference interaction was found (interaction T1 vs. T2: sum(*t*) = 739.28, *P*_cluster_ = 1; interaction T1 vs. T3: sum(*t*) = 2.11 × 10^3^, *P*_cluster_ = 0.45; interaction T2 vs. T3: sum(*t*) = 1.08 × 10^3^, *P*_cluster_ = 0.94). Based on our a priori hypothesis that if the ERP changes arising from mindfulness result of its attention training component then similar ERP changes should be observed in a control condition training those same attentional components, we performed follow-up post-hoc analyses to investigate group-specific changes over time. Follow-up pairwise comparisons revealed significant changes from T1 to T3 in the MT group at two distinct latency ranges (see Fig. 3a). Firstly, an increase in amplitude was observed in the early latency range spanning P1 latencies (33–107 ms) which was clustered unilaterally around left parieto-occipital areas (sum(*t*) = 7.13 × 10^3^, *P*_cluster_ = 0.01, *d* = 0.65, 33–107 ms post-cue onset). Secondly, we observed an increase in amplitude from T1 to T3 in the N1 latency range between 155 and 190 ms post-cue onset (sum(*t*) = − 5.6 × 10^3^, *P*_cluster_ = 0.045, *d* = 0.54, 155–190 ms post-cue onset). This effect was clustered around parietal–temporal regions in line with previous studies reporting ERP effects at N1 latencies^41^. Although this N1 effect did not reach statistical significance after 8 weeks of MT (sum(*t*) = − 4.26 × 10^3^, *P*_cluster_ = 0.10, *d* = 0.29, 145–194 ms post-cue onset), a progressive increase in amplitude during this latency range could be observed across the three timepoints (see Fig. 4a). Furthermore, no effect was observed between T2 and T3 (*P*_cluster_ > 0.05).

Follow-up pairwise comparisons for the CT group revealed a significant increase in amplitude from T1 to T3 between 178 and 235 ms post-cue onset (see Fig. 3b). This cluster spans N1 and P2 latencies, demonstrating increased amplitude of the negative N1 component followed by a concomitant reduction in amplitude of the following positive P2 peak (sum(*t*) = − 7.43 × 10^3^, *P*_cluster_ = 0.01, *d* = 0.73, 178–235 ms post-cue onset). This cluster was distributed over occipital regions at onset and later demonstrated a centroparietal distribution. While no significant clusters were observed from T1 to T2 or from T2 to T3 in the CT group (all *P*_cluster_ > 0.05), a progressive increase in amplitude during the N1–P2 latency range was observed over the three timepoints for the cue-stimulus A (see Fig. 4b).

These results indicate that at 6-month follow-up the MT group demonstrated an enhanced ability to mobilise neural resources at very early stages of information processing (P1 and N1 latencies), while the CT group demonstrated an enhanced ability to allocate neural resources in occipital regions during later attentional processing (N1–P2 latencies). These results were observed in response to the cue-stimulus A, which served as an alerting cue to the possibility of an upcoming target-stimulus X.

Therefore, in a next step, we applied the same analysis strategy to trial-averaged ERPs upon the target stimulus X. While we again observed a significant main effect of time (sum(*F*) = 1.72 × 10^4^, *P*_cluster_ = 0.019), no significant group (sum(*t*) = 7.47 × 10^3^, *P*_cluster_ = 0.07) or pairwise difference interaction (Interaction T1 vs. T2: sum(*t*) = − 2.4 × 10^3^, *P*_cluster_ = 0.5; Interaction T1 vs. T3: sum(*t*) = − 2.9 × 10^3^, *P*_cluster_ = 0.39; Interaction T2 vs. T3: sum(*t*) = − 257.23, *P*_cluster_ = 1). Thus, we performed follow-up pairwise comparisons to assess group-specific changes over time. Pairwise comparisons demonstrated a significant amplitude increase between 230 and 400 ms from T1 to T3 in the MT group (sum(*t*) = − 1.05 × 10^4^, *P*_cluster_ = 0.01, *d* = 0.36, 230–400 ms post-target X). The distribution of this cluster began in parietal regions, however by 300 ms the effect displayed a frontocentral distribution (see Fig. 3c). While this P3 effect reached significance only at T3, a progressive increase in amplitude during this latency period from T1 to T2 and from T2 to T3 was also observed (see Fig. 4c). No significant clusters were found in the CT group (maximal cluster: sum(*F*) = 3.29 × 10^3^, *P*_cluster_ = 0.56) for the target-stimulus X for any time point comparison (see Fig. 4d). These results indicate that at 6-month follow-up, the MT group demonstrated an enhanced ability to mobilise attentional resources in response to the target-stimulus X.

Taken together our results indicate that at 6-month follow-up the MT group demonstrated an enhanced ability to mobilise neural resources both at very early stages (P1 and N1 latencies) and later stages (P3 latencies) of information processing.

### Brain-behaviour correlations

**Figure 5:**
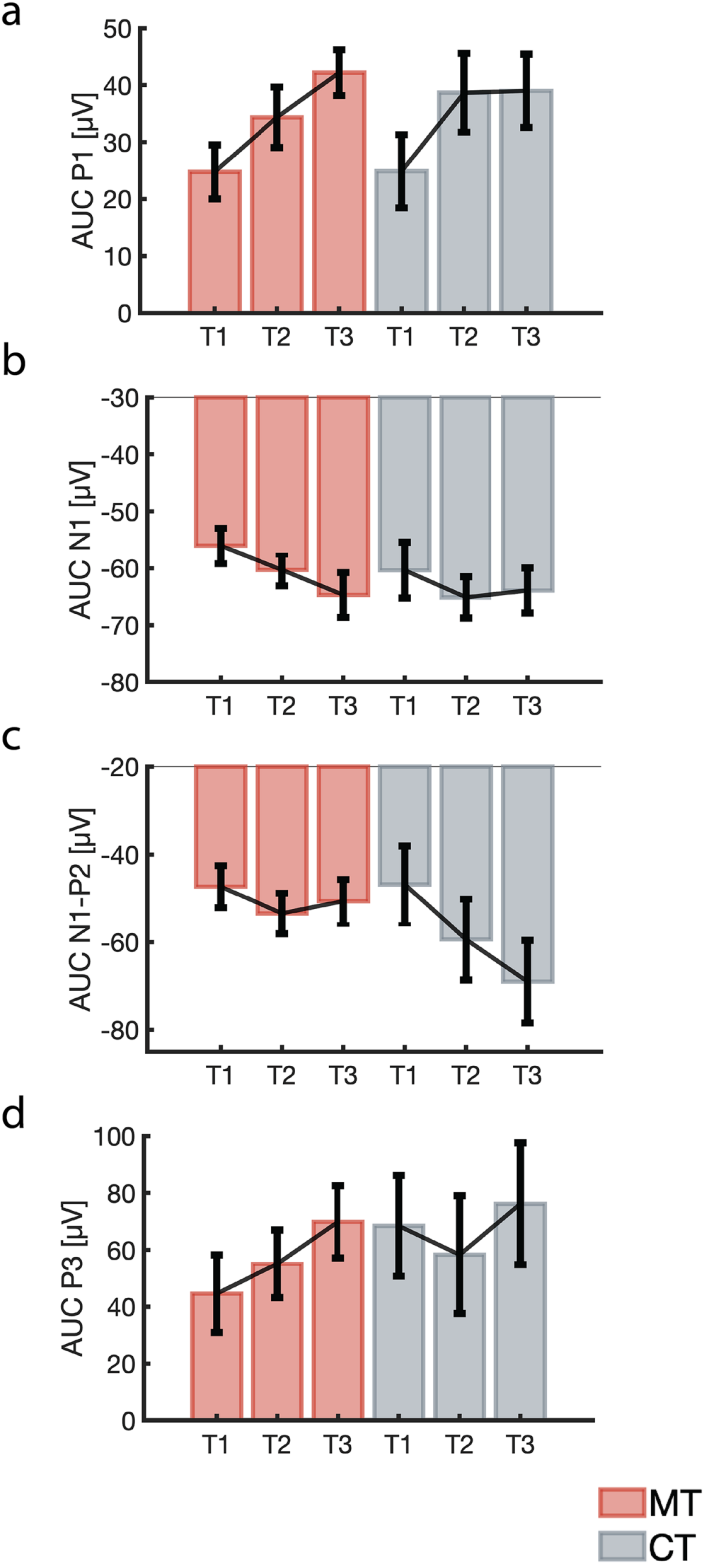
Area under the curve (AUC) for each significant cluster by group across time. (**a**) Plot of cue stimulus AUC P1 cluster by group for each timepoint (error bars show SEM). (**b**) Plot of cue stimulus AUC N1 cluster by group for each timepoint (error bars show SEM). (**c**) Plot of cue stimulus AUC N1–P2 cluster by group for each timepoint (error bars show SEM). (**d**) Plot of target stimulus AUC P3 cluster by group for each timepoint (error bars show SEM).

To determine if the differential neural changes observed within each group were related to behaviour, each ERP cluster was correlated with reaction time. We extracted the area under the curve (AUC) for each significant cluster across all time points within each group (see Fig. 5) and performed exploratory correlations with target RT as a primary behavioural outcome.

**Figure 6:**
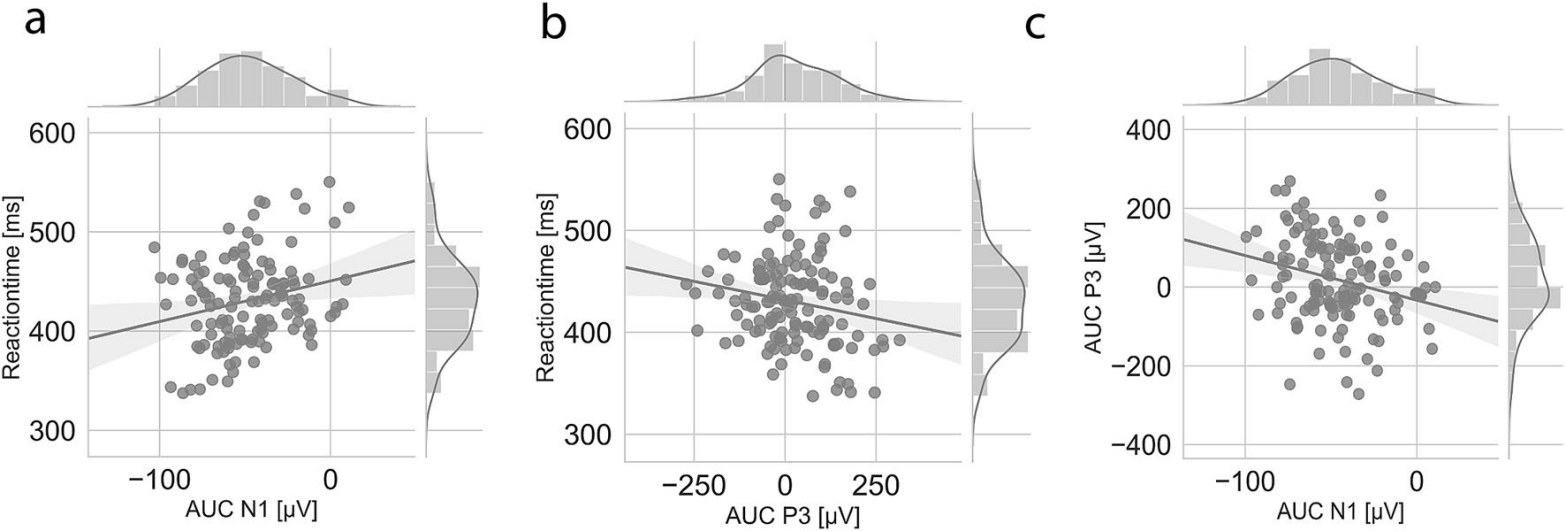
Brain-behaviour correlation analyses for the MT group. (**a**) Correlation and distribution of RT and cue AUC N1 activity for the MT group. (**b**) Correlation and distribution of RT and target AUC P3 activity for the MT group. (**c**) Correlation and distribution of cue AUC N1 and target AUC P3 activity for the MT group.

Among the ERP clusters identified within the MT group, a significant association was found between the AUC of the cue N1 effect (155–190 ms post-stimulus A) and target RT (*r*_(129)_ = 0.24, *p* = 0.007, CI [0.06, 0.39]), indicating that increasing N1 amplitude to the cue-stimulus is associated with shorter reaction times to the target-stimulus (see Fig. 6a). In addition, AUC of the target P3 effect (230–400 ms post-stimulus X) was negatively correlated with target RT in the MT group (*r*_(135)_ = − 0.19, *p* = 0.03, CI [− 0.34, − 0.02]), such that greater P3 amplitudes were associated with shorter reaction times (see Fig. 6b). No significant association between the AUC of the cue P1 effect (33–107 ms post-stimulus A) and target RT was found in the MT group (*r*_(138)_ = − 0.07, *p* = 0.44, CI [− 0.23, 0.10]). Within the CT group, no significant correlation was found between the cue N1–P2 effect and RT (*r*_(75)_ = 0.10, *p* = 0.38, CI [− 0.13, 0.32]). These results indicate that only the cue N1 and target P3 neural responses observed in the MT group were significantly associated with reaction time.

Since both N1 and P3 were related to RT in the MT group, we performed further analyses to determine if cue-induced neural activity in this group was related to target-induced neural activity. Correlational analysis revealed a significant association between cue N1 and target P3 (*r*_(132)_ = − 0.26, *p* = 0.002, CI [− 0.41, − 0.10]) in the MT group, such that increasing N1 amplitudes to the cue stimulus were associated with greater P3 amplitudes to the target stimulus (see Fig. 6c).

## Discussion

We examined attentional and neural changes in older adults after 6 months of mindfulness training to determine the ability of mindfulness to enhance processes known to decline in aging. Using a task sensitive to changes in the neural circuits subserving attentional control we found improvements in sustained attention in older adults that were maintained at 6-months follow-up. Contrary to our hypothesis, no significant ERP changes were observed immediately after the 8-week intervention. However, after 6 months of mindfulness training, participants demonstrated ERP changes clustered at left occipitoparietal sites indicative of enhanced sensory processing. These early neural changes were accompanied by increased activation at parietotemporal sites during perceptual discrimination, together with increased neural activation at centroparietal sites during post-perceptual processing of a subsequent target. Cue-N1 responses indexing perceptual discrimination were significantly correlated with P3 responses to targets requiring a response, supporting the functional role of this P3 response as reflecting the closure of a stimulus–response event^42^.

These changes suggest both an increased efficiency of the neural pathways subserving bottom-up visual processing together with an enhanced ability to mobilise top-down processes during perceptual discrimination after mindfulness training. During visual processing top-down attentional circuits begin to influence sensory processing within 30ms^43^, and are capable of modulating sensory neural activation as early as 60 ms^44^. Such early P1 attentional effects have been described as indexing a stimulus-triggered inhibition of task irrelevant networks to permit enhanced sensory processing through an increase in signal-to-noise ratio in task relevant networks^41,45^. The P1 attentional response therefore represents a form of early stimulus categorisation that precedes N1 processes. The N1 component has been shown to index perceptual discrimination, and serves to facilitate further task-relevant processing of the discriminated stimulus at longer latencies^46^. Attentional modulation of early P1 and N1 components demonstrates that the processing of sensory information is immediately affected by top-down processes as soon as it arrives in the cortex^44,47^. Furthermore, the speed with which this information arrives in the cortex has been shown to decline with increasing age due to deterioration of the white matter microstructure of the optic radiation connecting the thalamus to the visual cortex^17^. Our results suggest an enhanced efficiency within this bottom-up visual pathway following mindfulness training which may result from improved white matter microstructure integrity. Several studies have demonstrated widespread increases in white matter connectivity following long-term mindfulness practice^48,49^, suggesting that ongoing training may be capable of altering white matter microstructure leading to greater functional efficiency.

Our results suggest that these early sensory and perceptual changes may arise only after a prolonged period of mindfulness training, since such changes were not observed immediately after the 8-week program. This is consistent with findings from a cross-sectional study using practitioners with an average of 5 years of practice (*SD* 2 years) which reported enhanced sensory processing at P1 latencies clustered around left temporoparietal and temporo-occipital junctions, enhanced perceptual discrimination at N1 latencies clustered around middle occipital and middle temporal gyri, and enhanced P3 response at temporal and prefrontal sites^5^. This constellation of effects aligns precisely with the pattern of results reported here after 6 months of training, suggesting that improvements in early attentional processing may not take years of practice to manifest, but rather appear after 6 months of mindfulness training. While behavioural improvements in sensory processing and perceptual discrimination have been reported immediately after mindfulness training^3,50^, post-intervention neural effects at P1 and N1 latencies have not been found^12,14,29^. Thus, while a confluence of evidence indicates that mindfulness training may be effective at enhancing sensory processing and perception, such effects may not necessarily be evident immediately following an MBI.

Based upon these findings, it is reasonable to speculate that the ability of mindfulness to modulate early sensory processes may contribute to the reported therapeutic benefit of mindfulness-based therapies in treating various psychological disorders^51^. Increased neural responsiveness toward aversive stimuli has been observed in individuals with anxiety as early as 80 ms^52^, suggesting biased sensory processing of affective stimuli resulting in neural over-activation. Mindfulness training preferentially targets neural networks associated with attentional deployment in order to facilitate increasing levels of non-elaborative engagement with sensory experience devoid of cognitive and affective reactivity^9,53,54^. Our results together with those from cross-sectional studies of experienced practitioners demonstrate that mindfulness can modulate the initial sensory and perceptual processing of information. While it is possible that this modulation of initial sensory information may result in inhibition of irrelevant or affective processes, future studies are required to confirm this link. However, such an outcome is predicted by contemporary cognitive models of mindfulness which anticipate enhanced sensory and perceptual processing with increased proficiency, resulting in enhanced perceptual acuity wherein objects are apprehended in a manner concordant with their reality devoid of perceptual or cognitive biases^2^. As psychological disorders such as depression and anxiety involve distorted interpretations of experience^55^, the corrective modulation of sensory and perceptual processing that results from mindfulness training may contribute to its therapeutic benefit in the treatment of psychological disorders.

In addition to these cue-stimulus effects, the mindfulness trained group also demonstrated enhanced neural activation over centroparietal regions at P3 latencies during target processing. Like the cue effects, target P3 effects were present only after 6 months of training. This parietal P3 component has generally been considered to reflect contextual updating of memory processes^56,57^. However, recently it has been proposed that this posterior P3 instead reflects the completion of a stimulus–response event that bridges perceptual processing with response processing^42,58^. Under this model, monitoring processes akin to those involved in context updating are initiated in response to the stimulus and culminate with response onset, thereby completing the stimulus–response event. A role for P3 in linking perception and response via ongoing monitoring processes accords with our results. N1 responses to the cue stimulus were significantly correlated with P3 activity to a target requiring a decision response, suggesting a linkage between these processes. Furthermore, both N1 and P3 responses in this group were significantly correlated with response reaction time. In line with the P3 stimulus–response interpretation, it may be that the cue stimulus activated decision processes that only reached completion once a decision response was required toward the target. Therefore, since the mindfulness trained group displayed enhanced N1 discrimination of the cue stimulus which initiated the stimulus–response event, it is perhaps not surprising that similar enhancements in target P3 were observed as the stimulus–response event was completed. These findings suggest that mindfulness not only enhances sensory and perceptual processes as indexed by P1 and N1, but that these early effects influence later cognitive processes as indexed by the P3.

While the computer training group showed similar improvements in sustained attention performance, the ERP changes observed in this group were limited to N1-P2 discrimination of the visual cue, and this effect was not associated with reaction time. No ERP changes were observed in the computer training group toward the target stimulus requiring a response. Thus, while behavioural indices of sustained attention showed improvement, the only ERP effect in this group was reflective of enhanced visual discrimination concordant with visual computer-based game training. Since the CT participants engaged in slightly more training than the MT participants, we can conclude that the absence of similar ERP effects in the CT group was not due to insufficient training time. The higher level of daily training in the CT group is likely due to the fact that the game-based computer training condition was more engaging to participants than a sitting mindfulness practice focussed upon the breath.

It should be noted that ERP changes at N2 and P3 latencies have been reported immediately after cognitive training in older adults^25,27^, which suggests that such programs also have utility in targeting age-related cognitive declines. The fact that N2 and P3 changes have been reported immediately after both mindfulness and cognitive training programs in older adults suggests that similar effects could have been expected in the current study. While no immediate training effects were observed in the current study, inspection of the ERP waveforms at each cluster latency across the three timepoints clearly reveal a gradual progression toward the final 6-month effect. Thus, while these changes did not reach significance immediately post-intervention, it is clear that changes in neural activation were occurring. This may be due to both the MT and CT conditions being carefully designed to exclude additional intervention components which may otherwise have contributed to or confounded observed outcomes. The lack of intervention components such as relaxation, psychotherapy, or yoga which are commonly found in contemporary MBIs such as MBSR and MBCT enabled the accurate assessment of the effects of mindfulness in isolation from these factors. The lack of immediate training effects in the MT group may therefore be a function of the exclusion of these confounding factors, thereby enabling a more accurate assessment of the effect of 8 weeks of mindfulness training on neural activation.

Lastly, the contribution of practice effects in the present cannot be entirely ruled out. While frequent repetition of continuous performance tasks within a short time period has been shown to result in some reduction of reaction time and error rates^59,60^, reaction time coefficient of variation during sustained attention tasks has been shown to be virtually unaffected by practice effects^61^. Reaction time coefficient of variation is a robust measure of sustained attention and is sensitive to fluctuations in executive control performance. Given its resistance to practice effects, it is unlikely that the observed improvements reported in the current study are the result of practice effects. Rather, the improvements in reaction time coefficient of variation confirm the effectiveness of the training conditions to improve attentional performance. Importantly, a large body of evidence demonstrates that task-related ERPs show high levels of test–retest reliability and are resistant to practice effects over time periods utilised here^62–65^. Thus, while we cannot rule out practice effects in the absence of a no-training control group, the available evidence suggests that practice effects are unlikely to have contributed to the ERP effects observed in the MT group but not the CT group.

The current study demonstrates enhanced sensory and perceptual processing of visual stimuli after 6 months of mindfulness training. Together with gains in sustained attention and P3 neural responses, these changes indicate that mindfulness may have a role to play in slowing or reversing age-related declines in attentional processing. MRI studies examining long-term practitioners support the positive effects of mindfulness on brain aging^49,66^. The results of the current study suggest that rather than taking a lifetime to arise, these effects may manifest after 6 months.

## Materials and methods

### Participants and procedure

One hundred and twenty healthy adults aged over 60 years were allocated to the interventions (MT: *n* = 77; CT: *n* = 43) which were described to participants as attention training programs in order to blind them to experimental and control conditions. Participants were randomly allocated at a rate of 2:1 to the MT condition, as we expected a higher rate of attrition in this condition over the course of the study. No remuneration was provided, and all participants were screened for conditions and medications known to adversely impact cognitive performance as well as prior mindfulness or meditation experience. After participant attrition, data from 81 participants was available for all three timepoints (MT: *n* = 50; CT: *n* = 31). Demographic information for participants included in the analysis are presented in Table 1, while Supplementary Figure S1 presents CONSORT flowchart of participant retention and reason for drop out during the study period.

Participants completed either an 8-week mindfulness training (MT) or computerised cognitive training (CT) program. Both programs consisted of weekly group training sessions along with a daily home training component of 20 min/day in week one, increasing to 45 min/day at week eight. No on-going training requirement was set after the 8-week intervention, with participants choosing whether to continue the training exercises between the completion of the program and the 6-month follow-up assessment. Participants were however required to maintain a record of time spent engaging in the training exercises throughout the entire study period. Monthly group check-in sessions were conducted to maintain contact with participants and minimise attrition throughout the follow-up period.

EEG was recorded at each timepoint while participants completed a breath counting task followed by the AX-CPT. A breath counting task^35^ was used as a behavioural measure of mindfulness to assess the effectiveness of the MT intervention to enhance mindfulness skills in this mindfulness-naïve group. Prior to each assessment symptoms of anxiety and depression^67^ were assessed since they are known to adversely impact cognitive performance and EEG outcomes. No participant reported elevated symptoms of anxiety or depression. Participants completed the Wechsler Test of Adult Reading at T1 to derive an estimated full-scale intelligence quotient (FSIQ).

Ethical approval was granted by the University of the Sunshine Coast Human Research Ethics Committee (approval: HREC A-15–748). All procedures were conducted in accordance with the guidelines of the ethics committee and the Australian *National Statement on Ethical Conduct in Human Research*. Informed consent was obtained in accordance with the Declaration of Helsinki.

### Interventions

The MT program utilised a standardised mindfulness technique^2^ designed to activate the core cognitive processes recruited during mindfulness practice without the addition of secondary components which would otherwise limit the interpretation of outcomes. The MT program emphasised the development of unbiased, non-elaborative, and nonjudgmental attention to present moment experience, and began by cultivating mindful awareness toward the sensations accompanying the breath during a sitting exercise before expanding the scope to include all elements of participants’ experience. Participants were instructed to initially use a mental labelling technique (‘rising’, ‘falling’) to assist the orienting and maintenance of attention toward the sensations accompanying the breath. However, with increasing proficiency participants were encouraged to attend to these sensations without using the labelling technique, thereby facilitating the development of direct non-elaborative awareness. The technique requires self-regulating attention to minimise distraction and emphasises an unbiased, nonjudgmental quality of attentiveness toward participants’ present moment experience.

The CT program utilised a game-based format to target similar attentional processes to those activated during mindfulness practice. The program consisted of modified versions of standard psychological tasks including an Eriksen flanker task^68^, a visual search task^69^, a task-switching task^70^, a divided attention task^71^, a Corsi block task^72^, and a card matching task (see Supplementary Table S1 for tasks utilised in the CT program). Participants in the CT program performed a single task continuously each session to replicate the training of sustained attention that occurs in mindfulness practice. Tasks were changed weekly and were available to access online at any time. Both the CT and MT interventions were structurally equivalent and were matched for training contact time (see Supplementary Table S2 for weekly program content).

### Behavioural tasks

During the breath counting task participants were instructed to breathe naturally while being attentive to the sensations accompanying the rise and fall of the abdomen as they mentally counted the in–out cycles of their breath from one to 21 for 15 min. Participants reported each in–out cycle with a down-arrow key press on counts one to 20, while successful count cycle completion on count 21 was reported using a right-arrow keypress. Self-caught errors were indicated by a left-arrow key press. A piezo-electric crystal effort sensor placed around the chest tracked respiration rate to physiologically confirm breath counting accuracy. A breath counting accuracy score was calculated as a percentage of correct count cycles to total cycles attempted during the task (correct count/total cycles × 100), with self-caught errors recorded as incorrect count cycles.

The AX-CPT consisted of the serial presentation of angular shaped letters (A, E, F, H, L, N, T, V, X, Y, Z) presented in randomly allocated sizes (100, 120, 140, 160 or 180 points in black font) in the centre of a grey background for 200 ms. Following stimulus offset, a yellow fixation cross appeared for a randomly assigned inter-stimulus interval ranging from 500 to 1000 ms. Participants were instructed to respond as quickly and accurately as possible via keypress when the letter X appeared immediately following the letter A. The experimental block consisted of 800 randomly generated trials containing 60 AX target pairs (7.5% of trials), 60 A* pairs where * was a letter other than X (7.5% of trials), and 60 *X pairs where * was a letter other than A (7.5% of trials). Participants completed a practice block with performance feedback before commencing the experimental block to ensure task instructions were understood. Performance measures included total errors (percentage of errors to total trials), reaction time (RT), and reaction time coefficient of variation (RTCV; standard deviation/mean). Task instructions were presented on a monitor screen placed 65 cm from the participant. Stimuli were presented using E-Prime 2.0.10 software (Psychology Software Tools, Pittsburgh, PA. https://www.pstnet.com).

### EEG recording and analysis

EEG was recorded using a 128-channel BioSemi ActiveTwo system (BioSemi B.V., Amsterdam, Netherlands) with vertical and horizontal electrooculogram to monitor eye movements. Data was acquired at a sampling frequency of 1024 Hz. Preprocessing of EEG data was carried out in MATLAB version 2017b (Mathworks, Natwick, MA. https://au.mathworks.com) using the Fieldtrip toolbox version 20200810 (http://fieldtriptoolbox.org )^73^. Data was first segmented − 2000 ms before and 4000 ms after onset of either the cue-stimulus A or the target-stimulus X. Although the time period of interest occurred − 200 ms before to 1000 ms after stimulus onset, the data was padded to prevent edge artifacts from filtering and increase the number of samples to be embedded into the independent-component analysis (ICA) algorithm. In a second step, the data was filtered between 1 and 40 Hz using a zero-phase shift Butterworth IIR filter and referenced to the average of all channels. Visual inspection of the data for artifacts that might distort the outcome of the ICA algorithm was conducted and trials containing artefact were rejected. Following artefact removal ICA was used to remove eye-blinks, saccades, and any cardiac artefacts. The data was then visually inspected to identify residual artefacts not detected via ICA. Across all timepoints, less than ten percent of trials were rejected due to the presence of artefact. ERPs were obtained from correct trials segmented − 200 ms before to 600 ms after stimulus onset for both the cue-stimulus A and target-stimulus X. Trials were baseline corrected using the 200 ms pre-stimulus period and averaged to provide cue and target stimuli ERP responses.

### Statistical analysis

Independent groups *t* tests were performed to assess baseline between-group differences in age, FSIQ, breath counting accuracy scores, and AX-CPT performance measures. Between-groups difference in gender balance and handedness were examined using chi square analysis. Mixed model ANOVA with group (MT, CT) as a between-groups factor and time (T1, T2, T3) as a within-groups factors were performed for breath counting scores and AX-CPT measures. To quantify ERP effects we employed non-parametric, two-tailed cluster-based permutation tests with 5000 randomizations. Clusters were defined as spatiotemporal patterns (minimum number of simultaneously significant channels = 2) that exceeded the critical *t* value. The cluster mass was then extracted by taking the sum of all *t* values within this cluster. For each random partition, the maximum cluster (max sum of *t* values of all clusters within each random partition) was extracted, finally resulting in a distribution of 5000 maximum cluster statistics. The critical cluster threshold was set at alpha = 0.05. If the true (not-shuffled) cluster for the condition of interest was above the 95% cluster distribution, the cluster was considered significant. All cluster-based permutations were performed between 0 and 600 ms post-stimulus. Effect sizes using Cohen’s d were computed based on the significant clusters by averaging over significant channels and samples. To test whether significant ERP clusters were correlated with behavioral outcomes we extracted the area under the curve (AUC) for each significant cluster (significant channels and timepoints) using the function *trapz.m* in MATLAB and correlated this value with reaction time using Pearson’s correlation. Statistical analyses were conducted in Python version 3.7 (https://www.python.org) using the statistical package *Pingouin* version 0.3.7 (https://pingouin-stats.org)^74^. Statistical analyses were conducted by a researcher blinded to condition and study aims. For all data, a blinded 2-tailed (5th–95th percentile) automated algorithm was applied to detect outliers. False discovery rate calculated using Benjamini–Hochberg procedure^75^ was used to control for multiple comparisons, and only sub-critical threshold *p* values are reported.

## Supplementary Information

Supplementary Information

## Data Availability

Down-sampled EEG data and data analysis code is available at Harvard Dataverse (https://dataverse.harvard.edu/dataset.xhtml?persistentId=doi:10.7910/DVN/JT6LAQ). The raw non-resampled EEG data is available, upon reasonable request, from the corresponding author.
