# Supplementary material for: Neural changes in early visual processing after 6 months of mindfulness training in older adults
Source: Sci Rep. 2020 Dec 3;10:21163. doi: 10.1038/s41598-020-78343-w (PMC7713117; doi:10.1038/s41598-020-78343-w)
Supplement: Supplementary file 1 — Supplementary Information [file 41598_2020_78343_MOESM1_ESM.docx]

**Supplementary Information**

**Manuscript Title:**

Neural changes in early visual processing after six months of mindfulness training in older adults

Ben ISBEL ^a,b*,†^

Email address: bisbel@usc.edu.au

Jan WEBER ^c,†^

Email address: jan.weber@student.uni-tuebingen.de

Jim LAGOPOULOS ^a,b^

Email address: Jim.Lagopoulos@usc.edu.au

Kayla STEFANIDIS ^a,b^

Email address: kstefani@usc.edu.au

Hannah ANDERSON ^a,b^

Email address: handerson@usc.edu.au

Mathew J. SUMMERS ^a^

Email address: msummers@usc.edu.au

**Supplementary Figure S1**

CONSORT flowchart of participant retention and reason for drop out during the study period.


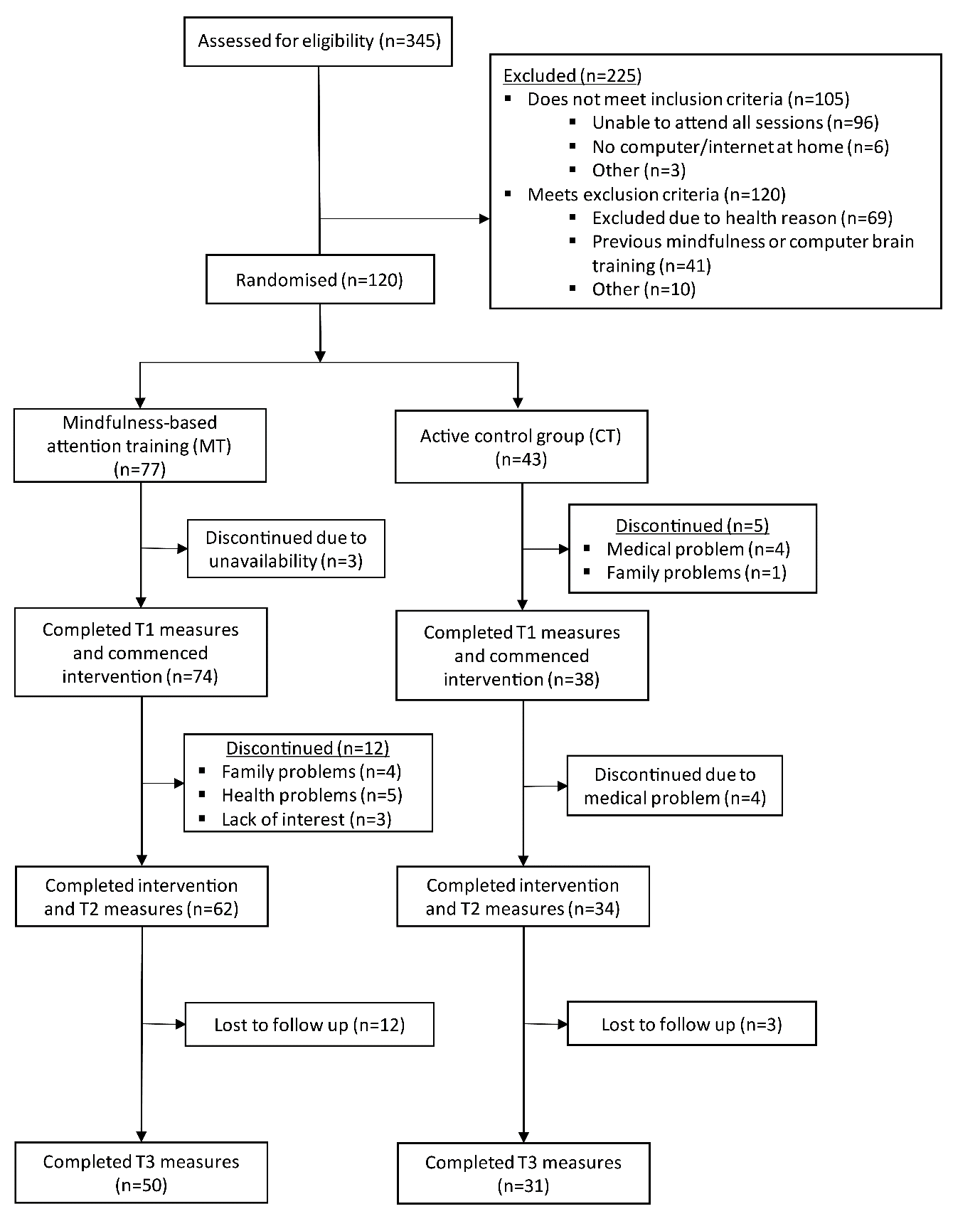


**Supplementary Table S1**

Tasks utilised in the active control condition

| Task | Description |
| --- | --- |
| 1. Flanker task based upon Eriksen and Eriksen (1974) | Participants are instructed to indicate the direction of a central arrow flanked on either side by two distractor arrows. |
| 2. Visual search task based upon Treisman and Gormican (1988) | Participants are instructed to indicate a unique object amongst a field of similar distractor objects. The number of distractors increases with successful performance, while their differences become less obvious. |
| 3. Task switching paradigm in accord with Kiesel et al. (2010). | This task involves arrows of two different colours moving across a screen. Arrows may point in the direction they are moving, or in a different direction. Participants are instructed to switch between indicating either the direction of movement of the arrow, or the direction that the arrow is pointing, depending on the colour of the arrows. |
| 4. Task switching paradigm in accord with Kiesel et al. (2010) | Participants are shown a card with a number and a letter on it. Participants are instructed to switch between indicating whether the number is an even number or if the letter is a vowel or not, depending upon the location that the card is presented. Presented at the top of the screen, participants are to indicate if the number is even. Presented at the bottom of the screen, participants are to indicate if the letter is a vowel. |
| 5. Stroop task based upon Stroop (1935) | Participants are shown two cards with two colour words written on them. The words and the colour of the letters change with each trial. Participants are instructed to indicate if the colour of the letters on the second card matches the meaning of the colour word presented on the first card. |
| 6. Moving visual divided attention task based upon Treisman (1982) | A number of identical objects move randomly within a fixed field. Participants are required to select one object at a time at three second intervals, without selecting the same object twice, until all objects have been selected. The number of objects increases with successful completion of the task. |
| 7. Static adaptation of the Corsi block task (Milner, 1971) to assess working memory (Baddeley, 2003) | Participants are presented with a grid of squares of a uniform colour. A small number of squares change colour briefly before changing back to the original uniform colour. Participants are instructed to indicate which squares changed colour after they have switched back to the uniform grid colour. The number of squares in the grid as well as the number of squares changing colour increases with successful performance. |
| 8. Serial working memory task (Baddeley, 2003) | Participants are presented with cards showing a variety of coloured shapes that appear serially. The new card remains face-up, while the card that appeared previously is turned face-down. Participants are instructed to remember if the card currently face-up matches the card that appeared two cards previous. |

Participant instructions:

*Each day play one game continuously for the session period. Do not take a break during the session. Keep playing the game for the duration of the session. Find a quiet place or room where you will not be disturbed. Set an audible timer so you do not have to check the time to know when the session is over.*

**Supplementary Table S2**

Weekly content for training interventions

|  | MT program | CT program |
| --- | --- | --- |
| Week 1 | *Introduction to program:*   - Overview of program and training requirements - Introduction to basic exercises | *Introduction to program:*   - Overview of program and training requirements - Introduction to basic exercises |
| Week 2 | *What is Attention?*   - Introduction to attentional selectivity and intensity - Arousal and attention - Controlling attention | *What is Attention?*   - Introduction to attentional selectivity and intensity - Arousal and attention - Controlling attention |
| Week 3 | *Working Memory:*   - What is working memory - Limits to working memory - Interference to working memory | *Executive Attentional Functions:*   - Inhibitory control and attentional interference - Introspection and attention |
| Week 4 | *Executive Attentional Functions:*   - Inhibitory control and attentional interference - Introspection and attention | *Working Memory:*   - What is working memory - Limits to working memory - Interference to working memory |
| Week 5 | *Age-related Cognitive Decline:*   - improving attention as we age - cognitive reserve | *Age-related Cognitive Decline:*   - improving attention as we age - cognitive reserve |
| Week 6 | *Emotional Regulation:*   - relationship between emotional control and attentional control | *Emotional Regulation:*   - relationship between emotional control and attentional control |
| Week 7 | *Metacognition:*   - Introspection - Developing insight into attentional performance | *Controlled vs Automatic Cognitive Processes:*   - how attention and effort relate to controlled vs automatic processes |
| Week 8 | *Controlled vs Automatic Cognitive Processes:*   - how attention and effort relate to controlled vs automatic processes | *Metacognition:*   - Introspection - Developing insight into attentional performance |
